# Editorial: Genetics and ontogeny in precision therapeutics for children

**DOI:** 10.3389/fgene.2023.1308169

**Published:** 2023-10-27

**Authors:** Feng Chen, Funda Tuzun, Catherine M. T. Sherwin

**Affiliations:** ^1^ Pharmaceutical Sciences Researches Center, Department of Pharmacy, Children’s Hospital of Nanjing Medical University, Nanjing, China; ^2^ Department of Pediatrics, Faculty of Medicine, Dokuz Eylül University, Izmir, Türkiye; ^3^ Department of Pediatrics, Boonshoft School of Medicine, Wright State University, Dayton, OH, United States

**Keywords:** precision medicine, pediatrics, pharmacogenomics, ontogeny, developmental pharmacology, drug-metabolizing enzymes, drug transporters

## 1 Background

The precision medicine framework aims to maximize therapeutic benefit by modifying treatment regimens based on changes in individual patient genomic, demographics, and environmental factors (Jameson and Longo, 2015). Such improvement in drug use could yield significant and even lifelong benefits in the pediatric population. On one hand, the importance of genetic factors to precision medicine is self-evident, reflected in the overwhelming research reports and the continuous attempts and efforts to promote individualized medicine (Sadee et al., 2023). As one of the critical elements of individualized drug therapy, pharmacogenomics obviously drives the practical clinical application of precision medicine. On the other hand, we cannot overemphasize the importance of growth and development for the pediatric population’s drug efficacy and adverse reactions. For drug-metabolizing enzymes (DMEs) and drug transporters (DTs), these proteins’ developmental changes in expression levels and activities drive age-specific pharmacokinetics (Kearns et al., 2003). Of note, genetics and ontogeny contribute to the observed individual variability in exposure and clinical response (McLaughlin et al., 2019). Therefore we ask the question, which of the two is more important? Ontogeny sometimes trumps genetics as a determinant of the enzymatic function in pediatric patients (Chapron, Chapron, and Leeder, 2022). Therefore, it is crucial to distinguish at what age ontogeny is the primary determinant and at what age genetic variation becomes predominant (Leeder, 2022). Consequently, to advance the clinical application of precision medicine in the pediatric population, it is not too late, and there needs to be a deeper look into the impact of genetics and ontogeny factors.

## 2 Scope

The aim of this Research Topic was to show the key considerations for implementing precision therapeutics for children. To this scope, we have collected reviews, research articles, case reports, and both pre-clinical and clinical study protocols that addressed: 1) Current literature reporting the “state-of-the-art” pharmacogenomics and ontogeny applied to pediatric pharmacology; 2) Effects of genetics and ontogeny factors of DMEs on individualized medicine in pediatrics; 3) Effects of genetics and ontogeny factors of DTs on individualized medicine in pediatrics; 4) Combining therapeutic drug monitoring (TDM) with genetics and ontogeny in pediatric precision medicine; 5) How to assess the influence of genetic and ontogeny factors in quantitative pharmacology and population pharmacokinetic (PPK) and pharmacodynamic studies; or 6) How to evaluate the influence of genetics and ontogeny factors in omics research.

## 3 Overview of contributions

Understanding the relationship between the genotypes and phenotypes of DMEs like CYP450 enzymes is highly helpful in comprehending individual variability in pharmacokinetics, particularly in children (Klomp et al., 2020). Indeed, studying the genotype and phenotype of DMEs in children remains challenging. In a recent study, Rodieux et al. have reported the use of CYP phenotyping and/or genotyping tests in children in a real-world setting and assessed the correlation between the predicted phenotype and the measured phenotype. Results revealed that the results of metabolic investigations could contribute to the clinical event in up to 60% of clinical situations, but a highly variable concordance existed between genotype and phenotype in children. Therefore, the authors conclude that the decision to utilize genotyping, phenotyping, or a combination of both should be evaluated on an individual basis, considering the limitations of each approach, the specific clinical question at hand, and the characteristics of the patient involved.

As well known, genetic variation as one of major factors contributes to those observed variability in drug disposition and response in children. For example, Ali et al. have evaluated the effect of genetic variants in *ITPA*, *TPMT*, *NUDT15*, *XDH*, and *ABCB1* on 6-mercaptopurine (6-MP) related toxicities like neutropenia in children with acute lymphoblastic leukemia (ALL) from Ethiopia. The findings indicated that specific genetic variations in *XDH* were linked to grade 4 neutropenia in individuals receiving 6-MP. Similarly, genetic variants in *ITPA* were associated with neutropenic fever in the same population and *XDH* rs2281547 was identified as a genetic risk factor for then grade 4 hematologic toxicities. Arguably, when using 6-MP, it is important to take into account genetic polymorphisms in enzymes other than *TPMT* and *NUDT15* that are involved in its pathway (Guo et al., 2022). This consideration might help prevent the occurrence of hematological toxicities.

In another case report, El Masri et al. presented a case study involving a 14-year-old patient who underwent high-dose methotrexate treatment and subsequently experienced acute fulminant liver failure and acute kidney injury. Through genotyping of *MTHFR*, *ABCB1*, *ABCG2*, and *SLCO1B1*, variations in all analyzed genes were identified. The findings indicated that the tested genetic variants predicted a reduced rate of methotrexate elimination, potentially contributing to the patient’s clinical outcomes. Therefore, pharmacogenetic testing could be a potential solution to prevent such adverse drug effects (Wang et al., 2022).

The importance of precision diagnosis based on pharmacogenomics in pediatric precision medicine is self-evident. For example, in a review article, Yen et al. have provided a comprehensive understanding of the impact of genetics and epigenetics on both short-term and long-term outcomes in Neonatal Abstinence Syndrome (NAS). They explored recent research endeavors that utilize polygenic risk scores to stratify NAS risk and investigate salivary gene expression as a means of understanding neurobehavioral modulation. Additionally, they also discussed those emerging studies focused on neuroinflammation resulting from prenatal opioid exposure, which uncovered new mechanisms and eventually contribute to the development of innovative precision therapeutic approaches for neonates in the future.

In the other case report, Xu et al. have presented the clinical manifestations and gene variants in 4 sporadic cases of 3M syndrome in Chinese children from different families. The authors identified several truncating mutations of *CUL7* and *OBSL1* in Chinese patients with 3M syndrome. Therefore, Xu et al. conclude that children who exhibit short stature, specific facial features, and physical symptoms should be referred for genetic testing in order to obtain an accurate diagnosis and precision therapeutics.

## 4 Conclusion

In conclusion, genetics and ontogeny do indeed influence the disposition of medications and potential clinical treatment outcomes in children, which can differ significantly from those in adults. The contributions to this Research Topic of Frontiers in Pharmacology and Frontiers in Genetics clearly demonstrate the value of genetics and ontogeny knowledge for precision therapeutics in pediatric patients. However, there are still numerous challenges that hinder the widespread implementation of these practices in routine clinical care. The assessment of the interaction between genetics and ontogeny poses significant challenges, and successful cases are relatively rare. Regardless, it is crucial to emphasize the significant role of genetics and ontogeny in pediatric precision medicine and to ensure that their potential impact is fully considered and ingrained in the minds of relevant healthcare professionals.
